# Glucose as fuel for HPA axis regulation: A narrative review on energy metabolism, stress reactivity, and early life adversity^⋆^

**DOI:** 10.1016/j.cpnec.2026.100374

**Published:** 2026-08-04

**Authors:** Jens C. Pruessner, Maria Meier

**Affiliations:** a Department of Psychology, University of Konstanz, Germany; b Centre for the Advanced Study of Collective Behaviour (CASCB), University of Konstanz, Germany; c Department of Child and Adolescent Psychiatry/Psychotherapy, University of Ulm, Germany

**Keywords:** Glucose metabolism, Cortisol, HPA axis, Stress reactivity, Early life adversity, Autonomic nervous system

## Abstract

Stress physiology and energy metabolism are inherently linked. While the physiological stress response mobilizes energy, early experimental work by Clemens Kirschbaum and colleagues demonstrated that glucose availability in turn shapes the stress response: Fasting attenuates cortisol reactivity, whereas glucose administration restores or amplifies it. Since then, an active line of research on this topic evolved, yet the role of glucose availability in shaping stress regulation still remains incompletely understood.

In this targeted, narrative review, we first synthesize evidence from the last 30 years that showed that blood glucose acts as a physiological modulator of acute HPA axis responsivity to stress. Second, we discuss these observations within the framework of the Selfish Brain Theory, which conceptualizes the endocrine stress axis and sympathetic nervous system as allocative systems that prioritize cerebral energy supply under conditions of acute stress. Third, we review potential consequences of the glucose-stress link in the context of stress occurring chronically or during sensitive developmental periods, such as early life adversity (ELA). Finally, we argue that future work should adopt a multisystemic perspective integrating more than just glucose as metabolic modulator as well as sympathetic, parasympathetic, endocrine processes alongside changes in affect. Here, we call for moving beyond controlled laboratory settings to investigate how energy metabolism and food intake shape stress perception in daily life. Such approaches may improve our understanding of how stress physiology and energy regulation jointly contribute to shaping the risk for metabolic and stress-related disorders.

## Highlights

• Glucose availability acts as a gatekeeper for cortisol stress reactivity.
• The Selfish Brain Theory frames stress systems as cerebral energy allocators.
• Early life adversity disrupts both glucose metabolism and HPA regulation.
• Future work should adopt multisystemic approaches and naturalistic settings.

## 1 Introduction

Stress response regulation and energy metabolism are tightly interconnected physiological processes [1]. Acute stress responses mobilize energy, while metabolic signals simultaneously shape how stress systems respond to environmental demands. Early work in rodents by Mary Dallman and colleagues provided first evidence for this bidirectional link: Her work showed that cortisol and insulin jointly regulate daily energy flow, and that the hypothalamic circuits governing energy balance directly modulate the responsivity of the hypothalamic-pituitary-adrenal (HPA) axis [2]. This metabolic feedback has later been discovered to dampen stress responses via metabolic feedback in the context of comfort food [1]. In light of this, the HPA axis is not merely a stress-reactive system that incidentally affects metabolism; it is, in part, a metabolically regulated system whose responsivity is gated by the organism's current energetic state [3]. Despite this close physiological link, research on the regulation of the HPA axis and research on metabolic regulation have to a great extent developed in parallel rather than in direct integration. In this narrative, we want to focus on one specific aspect of the stress-metabolism interaction that has received less attention in the human literature to date and builds on seminal work by Clemens Kirschbaum and colleagues, who this special issue is dedicated to: the role of glucose availability in modulating HPA axis responsivity to acute stress.

This manuscript is intended as a targeted, narrative, and opinion-driven synthesis rather than a systematic review of all literature linking metabolism and stress physiology. First, we summarize experimental findings in humans demonstrating that glucose availability influences cortisol responses to acute stressors in healthy adult populations. Second, we discuss how these observations can be conceptualized within the framework of the Selfish Brain Theory [4], which proposes that stress systems function partly to prioritize cerebral over peripheral energy supply. Third, we extend this perspective to the literature on effects of stress during sensitive developmental periods, in particular, early life adversity (ELA), which has been often linked to disturbances in both stress physiology and metabolic regulation [5,6]. We propose that alterations in glucose regulation, autonomic activity, and HPA axis responsivity may represent partially overlapping developmental adaptations rather than isolated phenomena. Lastly, we suggest directions we believe the field should pursue to better characterize how metabolic signals shape stress regulation and to elucidate the mechanistic links between energy homeostasis and the stress system.

## 2 The link between stress physiology and glucose metabolism

Many daily stressors across different domains (e.g., at work, in academic contexts) are of social-evaluative nature. That is, they present a threat to the *psychological integrity* of individuals and thereby trigger a cascade of physiological processes termed “stress response”. The activation of the sympathetic-adrenal-medullary (SAM) and the HPA axis thereby eventually triggers the release of epinephrine and cortisol [7] – two of the most prominent mediators in the “neuro-symphony of stress” [8]. While in the last decades, researchers in psychoneuroendocrinology have put much focus on the impact of psychological stressors on health,^1^ the stress response is primarily built to defend against environmental threats to homeostasis, such as injury or hypoglycemia [10]. Revisiting these origins may therefore offer valuable insight into the broader regulation of stress physiology, especially in the context of how metabolic signals modulate stress reactivity. Here, the significance of the stress system plays a crucial role when the most readily available fuel for our cells, glucose (used to generate adenosine triphosphate (ATP) [11]), is deficient in our system.

### 2.1 Hypoglycemia and counterregulatory stress physiology

The neuroendocrine response to hypoglycemia represents a complex, coordinated physiological process that strongly stimulates the stress response systems with the aim to restore glucose homeostasis if glucose levels fall below a certain threshold. This happens because the central nervous system (CNS) relies almost exclusively on glucose for its energy needs [12] and is a highly energy-consuming organ; at rest, the brain accounts for around 20 % of the body's total energy expenditure [13,14]. Further, as only a very limited amount of glucose can be stored in the brain in the form of glycogen, it is dependent on a constant supply from the periphery [13]. Therefore, maintaining sufficient blood glucose concentrations at all times is of high importance. Consequently, hypoglycemia is a serious – and potentially fatal – condition that is accompanied by a distinct constellation of psychological and neurocognitive symptoms [15,16]. These are categorized into neurogenic (autonomic) and neuroglycopenic symptoms. Neurogenic symptoms, such as nervousness and anxiety, are mediated by the activation of the SAM axis and act as essential precursors to the perception of a falling glucose level. As glucose deprivation in the brain intensifies, neuroglycopenic symptoms emerge, characterized by difficulty in thinking, confusion, disorientation, and tiredness. Further symptoms include difficulty concentrating, dizziness, and faintness. These symptoms follow a glucose threshold with autonomic warning signs appearing at around 58 mg/dL (3.2 mmol/L) of blood glucose levels, whereas significant cognitive dysfunction and more severe neuroglycopenic symptoms manifest at around 50 mg/dL (2.8 mmol/L). If the hypoglycemic state persists without correction, it can lead to a progressive alteration of consciousness, culminating in seizures, coma and death [11]. When blood glucose concentrations begin to decline due to reasons such as a lack of food or an excess of insulin secretion, the body initiates a hierarchical sequence of counterregulatory defenses to avert the progression of hypoglycemia and ensure a recovery of fuel availability [12].

The first response occurs at approximately 80 mg/dL (4.4 mmol/L), at which point the body begins to reduce insulin secretion [12,17]. As glucose levels decline further to around 68 mg/dL (3.8 mmol/L) the system triggers the release of glucagon and epinephrine [18]. Glucagon serves as the primary factor in immediate glucose correction by stimulating hepatic glycogenolysis and gluconeogenesis, while epinephrine provides a secondary, multi-organ response to sustain glucose levels [16]. The activation of the HPA axis alongside the release of growth hormone occurs as a last defense when glucose levels continue to drop below these thresholds. This step is initiated at a plasma glucose threshold of approximately 58 mg/dL (3.2 mmol/L) [12]. In contrast to the immediate effects of glucagon and epinephrine, cortisol and growth hormone induce metabolic changes over longer periods, typically several hours [18]. The primary function of cortisol in this context is to raise blood glucose levels by stimulating gluconeogenesis in the liver and lipolysis in adipose tissue. While growth hormone and cortisol are not critical for the immediate recovery from acute hypoglycemia [19], they play a vital role in defending against prolonged hypoglycemic states [18]. These hormones are thus essential for limiting the degree of hypoglycemia during extended periods of energy deprivation.

This complex response is integrated and coordinated by the CNS, which acts as the master regulator [20,21]. Specialized glucose-sensing neurons located in the periphery (such as the portal vein) and within the brain (primarily the hypothalamus and hindbrain) detect variations in glucose concentration [20]. Specifically, networks in the ventromedial hypothalamus (VMH) and the arcuate nucleus process this information and relay signals to motor neurons that trigger the neuroendocrine output of the adrenal cortex and other glands [13,20]. This redundancy in counter-regulation, where multiple factors are secreted within a narrow glycemic threshold, provides a failsafe system to protect the brain from neuroglycopenic damage. This example highlights the importance of the stress-reactive systems to prevent blood glucose levels to fall below life-threatening thresholds.

### 2.2 Glucose availability as a modulator of endocrine stress reactivity – early studies by Clemens Kirschbaum and colleagues

Building upon the fundamentals of the hypoglycemic response as well as the insulin-antagonistic and gluconeogenetic effects of cortisol, an (at least implicit) expectation was that of an inverse association between glucose availability and cortisol. Since HPA axis activation serves to provide the body with energy, low energy levels should lead to a greater activation, so that the HPA axis can provide more of the needed fuel by longer and more sustained gluconeogenesis in the liver. Kirschbaum and colleagues were among the first to challenge this traditional assumption in pioneering studies by revealing instead that the availability of glucose was a prerequisite for a robust HPA axis stress response [22]. In other words, energy availability serves as a critical gatekeeper for HPA axis activation in the context of stress. In a first study, Kirschbaum and colleagues conducted two experiments to investigate the impact of short-term fasting compared to glucose administration on adrenocortical responses. In study 1, healthy men fasted for 8-11 h and were then given either 100 g of glucose or water 1 h before being exposed to the Trier Social Stress Test (TSST), resulting in the activation of the HPA axis. In study 2, habitual smokers underwent a similar fasting protocol but were stimulated via nicotine consumption (smoking) rather than a psychosocial task. In both experiments, fasted participants who received only water failed to mount a significant cortisol response to the stimulations [22]. Conversely, participants who received a glucose load showed a robust increase in free cortisol levels following the TSST or nicotine consumption. Importantly, glucose alone did not trigger a cortisol surge, highlighting that it was the interaction of glucose with HPA axis stimulation that produced this result. Also, the fasted participants in the TSST group still exhibited significantly elevated heart rates, indicating that the lack of cortisol was not due to a lack of psychological arousal, but rather a specific inhibition of the HPA axis [22]. To determine if this effect was specific to glucose or a general response to caloric intake, the group expanded the design in another study [23]. Here, they compared the effects of glucose, protein, fat, or water administration in fasted men on stress responses to the TSST. This study demonstrated that specifically glucose load amplified the cortisol response. Protein and fat administration failed to restore HPA axis reactivity, with cortisol levels in those groups remaining as blunted as those who received water [23].

These results presented a somewhat counterintuitive finding in the context of classical stress theory. Traditionally, cortisol was viewed as a hormone that rises during stress to mobilize energy particularly when needed. Thus, one could expect that during a low-energy state (fasting), the body would be *more* inclined to trigger an HPA response to secure fuel. Instead, these studies found that low blood glucose levels lead to a decrease or abolition of cortisol stress responsivity, and that high blood glucose levels amplify the cortisol response to both psychosocial and pharmacological triggers.

At the time, the authors proposed several possible explanations for this glucose-dependent modulation. First, Kirschbaum et al. [22] hypothesized a permissive effect, where the “ready access to energy” is a physiological prerequisite for mounting an acute stress response. From a metabolic standpoint, the synthesis and secretion of hormones require adenosine triphosphate (ATP); thus, the brain may inhibit these processes when fuel is scarce to prioritize survival. Secondly, authors suggested a serotonin associated effect, where glucose intake triggers insulin secretion, which facilitates the transport of tryptophan into the CNS. This increases serotonin synthesis, a neurotransmitter known to have a stimulatory effect on the HPA axis at the hypothalamic level. Thus, the amplification of the HPA axis response might be an indirect consequence of serotonin. Finally, in the second study [23], the authors emphasized specific CNS mechanisms over peripheral ones. Referencing the rodent work by Mary Dallman's group, they suggested that the HPA axis might be under the control of CNS centers that monitor energy availability, particularly the VMH [24]. As pointed out earlier, this structure contains glucose-sensing neurons and high glucose/insulin levels stimulate the VMH, providing a permissive input to the paraventricular nucleus (PVN) of the hypothalamus, which is the master regulator of the HPA axis. Conversely, low glucose levels could inhibit the VMH/PVN, thereby “shutting down” the HPA axis' ability to respond to external stimuli.

In summary, the authors suggested that glucose might have a permissive effect on HPA axis responsivity [22,23]. It became clear from these hallmark studies that the axis requires a signal of sufficient glucose availability before it will commit to the energetic “expense” of activation, while the exact mechanisms remained to be empirically demonstrated. Although these early studies involved relatively modest sample sizes by contemporary standards, which likely overestimated effect sizes (cf. [25]), they provided important proof-of-concept evidence that stimulated a broader line of research on metabolic modulation of stress physiology. More recent studies have generally supported the robustness of the glucose-related HPA axis modulation, while extending the investigations into stress-associated variables.

### 2.3 Recent extensions of the glucose-stress literature

As research in the field has matured, studies have moved on from establishing the glucose-cortisol link to exploring its boundaries, including the effects of shorter fasting periods, the role of psychological expectations, and the potential impact on cognitive functions like memory. From personal conversation we know that, since the hallmark studies by Clemens Kirschbaum and colleagues, some laboratories started to provide grape juice or dextrose prior to stressor exposure to boost the cortisol response. More systematically, a research group in Regensburg set out to study whether this change in procedures indeed triggered the hypothesized effect. Specifically, the group tested whether the cortisol amplification following glucose load generalized (1) across shorter fasting windows that are typical in stress research (i.e., 2 to 4 h), (2) across men and women, and (3) across different types of sugary drinks [26]. Tapping into the question of whether the sweet taste of glucose plays a role in this context, they explored the effect of maltodextrin, a less sweet, yet caloric oligosaccharide with similar glycemic index as glucose. They invited 103 healthy participants after a 3-h fast who either consumed 200 mL of grape juice containing 32 g of sugar, 75 g of glucose, 75 g of maltodextrin or nil 50 min before being exposed to the TSST. The results indicated an increased cortisol stress response following glucose and grape juice consumption, with effects being stronger in males compared with females. Interestingly, maltodextrin did not have the same effect as glucose and grape juice, despite its similar caloric content. This suggested that the effect is still relevant even in shorter fasting windows, may be more pronounced in male participants, and may be influenced by both the sweetness and the calories that glucose provides.

To examine whether the glucose-induced HPA boost generalizes across different types of challenges, a research group in Trier investigated the effects of 75 g of glucose versus an artificial sweetener (cyclamate and saccharine) and water on stress reactivity in a sample of 151 healthy men [27]. Similar to the shorter fasting window used in the Regensburg study [26], participants fasted for only 4 h, a timeframe common in laboratory stress studies conducted in the afternoon. The drinks were consumed approximately 30 min prior to stressor exposure. Participants were exposed to either the TSST or the Cold Pressor Test (CPT) to compare the effects of glucose on psychosocial against physical stress. The results confirmed a significant but small effect of glucose in amplifying the cortisol response compared to water, even after this shorter fast. Interestingly, this effect appeared more pronounced during the psychosocial stressor (TSST) than the physical one (CPT). Because the sweetener group did not show a similarly pronounced response, the authors concluded that – in healthy males – the effect is not driven merely by the sweet taste, but rather by the metabolic availability of glucose [27]. Similar to the early studies by Kirschbaum, the group found no evidence for an effect of glucose on autonomic stress reactivity (as assessed via heart rate and salivary alpha amylase). In contrast to the earlier studies, they found no evidence for a linear correlation between blood glucose and cortisol. For the first time, they showed that glucose did not alter stress perception, which is in line with results showing that physiological and subjective stress responses are only weakly associated [28].

Our own group expanded this work by focusing on healthy women and investigating whether the effect was driven by caloric content (energy load), sweet taste, or psychological expectations [29]. We recruited 152 women who underwent an 8-h overnight fast and introduced an “energy prime”, which was a deceptive instruction in which participants were told their drink was either caloric or non-caloric, regardless of its true caloric content. Participants consumed either 25 g of saccharose, a non-caloric sweetener (stevia), or water approximately 30 min before being exposed to a modified group TSST [30]. The main analysis revealed that both sugar and sweetener increased cortisol stress reactivity compared to water, suggesting that – in healthy women – sweet taste itself might play a role in HPA modulation. However, a sensitivity analysis showed that sugar load led to the most robust boost in cortisol trajectories, thus resembling previous results in men [27]. Crucially, the energy prime (expectation manipulation) had no effect on the stress response, suggesting that biological signals outweigh psychological beliefs in this context. Confirming previous findings [27], we found no evidence for a modulation of the subjective stress response by sugar, and no systematic association between the drink-induced blood glucose increase and the magnitude of the cortisol response, suggesting that other moderating or mediating factors might also play a role here [29].

More recently, Rüttgens and Wolf [31] sought to determine if the glucose-related boost in cortisol has actual cognitive consequences, that is, if it modulated working memory and long-term memory (LTM) retrieval. They invited 72 participants after a 6-h fast and allocated them to consume either 75 g glucose or sweetener (stevia) before exposing them to the Socially Evaluated Cold Pressor Test (SECPT) that combines psychosocial stress elements with physical stress [32]. They neither found evidence for an amplified cortisol stress response following glucose consumption, nor modulating effects on working-memory and LTM [31]. Since the glucose boost has been shown more robustly in studies using the TSST as opposed to physical stressors [27], our group aimed to extend this work by utilizing a psychosocial stressor (TSST) and by testing effects on LTM of the stressful episode and a wordlist after a nightly consolidation [33]. In a two by two study design, 62 fasted adults consumed either 75 g of glucose or water and were exposed to either a stressful TSST with objects being placed in the room, or a friendly control task. We tested memory for stress-relevant information (objects handled by the committee) and stress-irrelevant information (a wordlist encoded at the cortisol peak). The results successfully replicated the glucose-induced boost of the HPA axis. Cognitively, we found that stress enhanced the memory of central, stress-relevant objects, and emotional words were generally remembered better than neutral ones. However, glucose availability did not modulate these memory effects; there was no interaction between the amplified cortisol response and memory performance. This suggests that while glucose intake consistently enhances the endocrine response to psychosocial stressors, it may play a less decisive role in how stressful episodes are encoded in LTM [31,33]. In a secondary analysis, we further found no supportive evidence that glucose and cortisol reactivity interactively affected food craving following stress exposure [34]. Interestingly, heart rate recovery from the TSST was impaired following glucose as compared with water ingestion [33], a finding suggesting that – in this study – glucose also modulated ANS stress regulation. Altogether, these studies collectively suggest that the amplification of HPA stress reactivity by glucose is a robust but complex physiological phenomenon.

Consolidating the aforementioned findings, a recent systematic review and meta-analysis by Kördel, Meier, Kühnel, and Kroemer [35] synthesized data from 21 studies involving 1216 participants to quantify how metabolic and hormonal states shape HPA axis reactivity. Eight studies comprising 458 participants focused on the effect of glucose on stress reactivity (see narrative summaries above). The analysis confirmed a robust modulatory role of glucose availability, finding that glucose administration was associated with a significant increase in cortisol responses to acute stress compared to fasting or non-glucose control conditions, which was described as small (*d* = .30) effect. This provides meta-analytic support for the notion that glucose intake boosts cortisol reactivity, while fasting tends to blunt it. In contrast, the effects of sex hormones, specifically progesterone and estradiol, on cortisol reactivity were found to be smaller, more variable, and overall inconsistent across the existing literature [35]. These findings suggest that metabolic signals may play a more direct or stronger modulatory role in shaping HPA axis regulation than fluctuating sex hormones. Despite the consistency of the glucose effect, the review also underscored that individual changes in circulating blood glucose levels did not correlate linearly with the magnitude of the cortisol increase, suggesting that blood glucose concentrations alone do not fully explain the HPA amplification.

Taken together, the recent body of literature confirms the initial findings by Kirschbaum and colleagues [22,23]. Key conclusions at this stage include that first, the effect is small [35], but pervasive: Glucose at various levels (i.e., 25 g up until 100 g) and at different temporal time lags before the stressor (i.e., 30 to 60 min) boosts the HPA axis reactivity in both men and women and after shorter (i.e., 3 to 4 h) and longer (i.e., overnight) fasts. Secondly, the lack of a consistent linear correlation between blood glucose rises and cortisol responses suggests that the mechanism likely involves insulin sensitivity or central glucose-sensing pathways in the hypothalamus rather than a simple peripheral energy signal [27,29,33]. Here, future studies might want to investigate other possible factors in more depth, e.g., insulin sensitivity or the role of other metabolic markers, such as ghrelin or triglycerides [35]. Third, while glucose amplifies the HPA axis stress response, this physiological boost does not appear to extend into enhanced memory for the stressful event [33] or increased food craving following stress [34]. In contrast, these studies provided little evidence that glucose modulated autonomic stress reactivity in heart rate or salivary alpha amylase, or that individuals perceived the stressor differently in terms of glucose load (in terms of subjective stress, arousal or pleasure ratings). This might be due to the fact that the studies were rather heterogeneous in their methodological approaches (cf. Table 1). Nonetheless, they pointed towards important potential modulators of the glucose-stress effect: First, this comprises biological sex, with possible differences in the effect of sweet taste in females vs. males [27,29] and a stronger effect in males as compared with females [26]. This may be related to sex-related differences in glucose metabolism and insulin sensitivity that also has been shown to vary across sexes and the menstrual cycle phase [36] – variables that have not yet been explored systematically in this context. Second, the nature of the stressor, and stressor intensity respectively, may modulate the effect, with more robust effects in moderate vs. mild and psychological vs. physical stressors [27,31,33]. Interestingly, no clear pattern on effects of fasting duration (short vs. long fast), lag between glucose intake and stressor (short vs. long), caloric load (low vs. high load) or time of day of testing (morning vs. afternoon) has been revealed so far, warranting further investigations that test the context-dependence more thoroughly. In summary, while the driving mechanism underlying the amplification of the cortisol stress response by glucose requires further elucidation, the growing body of literature continues to improve our understanding of the HPA-metabolism interface.

**Table 1:** Methodological comparison of published studies that investigated the effect of glucose administration on the acute stress response. TSST = Trier Social Stress Test. CPT = Cold Pressor Test. TSST-G = Trier Social Stress Test for Groups. SECPT = Socially-evaluated Cold Pressor Test.

Study	Sex	Fasting duration	Substance	Glucose dose	Stressor	Time lag between ingestion and stressor onset
Kirschbaum et al. [22] Study 1	Male	8-11 h	Glucose vs. water	100 g	TSST	60 min
Kirschbaum et al. [22] Study 2	Male	8-11 h	Glucose vs. water	100 g	Nicotine (smoking)	60 min
Gonzalez-Bono et al. [23]	Male	8 h	Glucose vs. protein vs. fat vs. water	100 g	TSST	60 min
Zänkert et al. [26]	Male and Female	3 h	Glucose vs. grape juice vs. maltodextrin vs. no drink	75 g (glucose and maltodextrin) 32 g (grape juice)	TSST	50 min
von Dawans et al. [27]	Male	4 h	Glucose vs. sweetener vs. water	75 g	TSST and CPT	30 min
Bentele & Meier et al. [29]	Female	8 h	Grape juice vs. water	64 g	TSST-G (modified)	20 min
Meier et al. [29]	Female	8 h	Saccharose vs. sweetener vs. water	25 g	TSST-G (modified)	20 min
Rüttgens & Wolf [31]	Male and Female	6 h	Glucose vs. sweetener	75 g	SECPT (cold pressor + social evaluation)	30 min
Meier et al. [33]	Male and Female	5 h	Glucose vs. water	75 g	TSST-O	20 min

## 3 The Selfish Brain Theory as an integrative framework of glucose-stress link

The summarized findings of how glucose shapes HPA axis responsiveness raise an important conceptual question:

*Why would the HPA axis require glucose before mounting a full stress response if one of cortisol's primary functions is itself to* support *energy mobilization?*

One theoretical framework that offers a potential explanation is the Selfish Brain Theory by Peters and colleagues [4]. This hypothesis posits that the brain occupies a primary, hierarchical position in energy metabolism, behaving in a “selfish” manner to maintain its own supply with energy when competing for resources with the periphery [4]. Central to this theory is the observation that the brain's energy supply must be preserved at all costs [13]. Historical data, such as that from Krieger [37], showed that during periods of starvation, integrity of the human brain – even though it is a highly energy-demanding organ [13,14] – is remarkably preserved, while other organs like the heart, liver, and kidneys can lose up to 40 % of their mass. This suggests the existence of a “brain-pull” mechanism, i.e., a physiological demand system that enables the CNS to secure its fuel irrespective of the peripheral energy status [38]. The term “selfish brain” is derived from the notion that the brain must manage a fundamental biological conflict: ensuring glucose availability for itself when somatic cells are competing for the same fuel [4]. According to the Selfish Brain Theory [4,39], the solution lies in the differential transport mechanisms of glucose into cells. Cells in the periphery, on the one hand, are dependent on insulin: the uptake of glucose into cells requires the activation of GLUT4 transporters by insulin. The brain, on the other hand, is insulin-independent: Glucose transport across the blood-brain barrier and into astrocytes is mediated by GLUT1 transporters, which function independently of insulin [13]. Thus, because the brain does not require insulin to “pull” glucose from the blood, it can secure its supply most effectively by preventing GLUT4 transporters from doing their job, that is, pulling glucose into somatic cells. By limiting the body's ability to sequester glucose via GLUT4, the brain can ensure that a higher concentration of circulating glucose remains available for its own insulin-independent GLUT1 transporters.

According to the Selfish Brain Theory [4,39], the primary tool the brain uses to achieve this glucose allocation is a mechanism referred to as Cerebral Insulin Suppression [39,40]. Under conditions of stress, which increase the brain's energy need significantly, the CNS activates the sympathetic nervous system (SNS) and the HPA axis. Here, stressful stimuli trigger activation of distinct structures at neuroendocrine relay stations, including – again – the VMH and PVN of the hypothalamus [7]. On the one hand, this results in the release of epinephrine and norepinephrine, both correlates of SNS activation. On the other hand, the HPA axis gets activated, leading to a release of corticotropin-releasing hormone (CRH) from the hypothalamus, adrenocorticotropic hormone (ACTH) from the anterior pituitary, and finally cortisol from the adrenal cortex [7]. These hormones directly interfere with the ability of GLUT4 to transport glucose: Epinephrine and norepinephrine but also cortisol directly inhibit insulin secretion from the pancreatic beta cells. Here, a well-designed experiment demonstrated that a higher increase in cortisol during stress was associated with a steeper increase in blood glucose levels without a corresponding rise in insulin [39]. When stressed participants consume carbohydrates, they typically experience postprandial hyperglycemia that is, their glucose levels after the carbohydrate meal are higher than in a non-stressed control group. However, despite these elevated blood glucose levels, serum insulin concentrations did not rise as they would under non-stress conditions [39]. This insulin suppression is mediated by norepinephrine and epinephrine and maintained through cortisol to ensure that glucose is not dissipating into peripheral storages in muscle or fat. As a consequence, glucose remains in circulation for a longer period, effectively allowing GLUT1 transporters to access more energy to satisfy the brain's heightened metabolic demand. Thus, according to the Selfish Brain Theory [4,39], the HPA axis does not merely react to stress; one of its core functions is to serve as an allocative “brain-pull” mechanism that safeguards the brain's energy supply at the expense of the body [40]. In turn, this may offer an explanation on the phenomenon of reduced cortisol stress responses at times of low glucose levels: with no glucose in circulation, there is no “need” for a strong cortisol secretion as no glucose could be transported into the brain [41]. Put differently, insulin is secreted when glucose levels rise to allow glucose to enter somatic cells, whereas cortisol is secreted when the brain's demand for energy is high (under conditions of stress) *and* glucose is available; in that sense, cortisol could be considered *the insulin of the brain* [4,40]. In light of this, the Selfish Brain Theory could be interpreted as a teleological extension of Dallman's mechanistic observations [2,42].

Beyond the prediction of glucose-stress interactions in acute stress situations, the Selfish Brain Theory further may explain how – under conditions of severe, chronic, or sustained stress – the system gets dysregulated and prone to store excess energy in the periphery (in the form of visceral fat) to adopt to the situation. This trajectory has direct clinical relevance: it is well documented that chronic or traumatic stress, particularly when experienced during sensitive developmental periods such as childhood and adolescence, substantially increases the risk for both psychopathology and somatic disorders, including metabolic disease [[43], [44], [45]]. Thus, in the following section, we consider how the acute glucose-stress interactions described above may translate into lasting dysregulation of both metabolic and stress systems when stress is experienced in early life.

## 4 The glucose-stress link in the context of early life adversity

To further refine our understanding of how energy metabolism and stress regulation intersect, we would like to turn to an example in which both are frequently disturbed together: early life adversity (ELA). ELA is an umbrella-term describing a broad spectrum of traumatic and adverse experiences during critical development periods such as childhood and adolescence that compromise the individual's security or safety, thereby inducing systematic and prolonged stress responses [6,46]. These experiences range from low socioeconomic status, growing up in a dysfunctional household, to victimization, bullying to physical or sexual abuse [46]. They may occur within the context of the family of origin, with primary caregivers playing a special role as both a potential source or buffer against ELA [47], or in other contexts like school or leisure activities.

### 4.1 Metabolic disturbances in the sequelae of early life adversity

Experiencing ELA is associated with a life-long increase in the risk for mental and somatic illness [5,6] and premature death [48]. Next to disturbances in stress physiology [6], ELA is associated with significant metabolic disturbances that include a heightened risk for obesity, dyslipidemia, type 2 diabetes, and a chronic inflammatory phenotype [5,[43], [44], [45],[49], [50], [51]]. For example, individuals with a history of ELA have a 40-60 % higher risk to develop severe obesity (body mass index > 35 kg/m^2^) as compared with those without such experiences [52]. Also, ELA is associated with an increased risk for the metabolic syndrome [45], a precursor of diabetes, characterized by a cluster of conditions including impaired glucose metabolism, central obesity, and hypertension [53]. Importantly, type, timing, severity and chronicity of ELA may differentially shape neurobiology [54,55] as well as the risk for pathology [54,56]. Accordingly, the mechanisms linking ELA, HPA axis regulation, and metabolic outcomes are unlikely to be uniform across individuals or contexts.

These metabolic disturbances are also reflected in altered circulating levels of metabolic hormones and inflammatory markers. Specifically, ELA is associated with an increased leptin to adiponectin ratio; while leptin (which signals levels of stored energy in the form of fat) is elevated [57,58], adiponectin (which has insulin-sensitizing effects) is decreased, promoting insulin resistance, one of the symptoms of the metabolic syndrome [58]. The association between ELA and increased leptin levels has also been supported in a recent longitudinal study that followed middle-aged women over 2.5 years [59]. Furthermore, ELA has been linked to increased levels of irisin, a myokine involved in glucose metabolism and energy expenditure, and elevated inflammatory markers, e.g., fibrinogen, C-reactive protein, and interleukin-6, which are considered cardiometabolic risk factors [58]. In sum, these multisystemic neurobiological dysregulations following ELA manifest in an increased allostatic load [60]. As these disturbances frequently co-occur with alterations in HPA axis regulation [6,61,62], this raises the question of how the two are linked.

### 4.2 Mechanisms linking early life adversity to metabolic dysregulation

Multiple biological, behavioral, cognitive, and social mechanisms may collectively underlie this relationship, including epigenetic processes, changes in brain structure and function, and social environmental influences [5,6]. A commonly found position in the literature is that disturbances in HPA axis regulation mediate the link between ELA and metabolic disturbances later in life [63]. This view indirectly proposes that metabolic disturbances are the *consequence* of stress system dysregulations. Of note, the inverse direction, or a parallel process feeding back and forth between the systems, is certainly also plausible. In fact, the development of the neuroendocrine and metabolic system in early life are not independent from each other. In a review synthesizing preclinical and clinical evidence, Yam and colleagues [64] argue that both, ELA and early life malnutrition produce strikingly similar long-term alterations in HPA axis reactivity and emotional function, suggesting a shared mechanistic pathway rather than independent routes to dysfunction [64]. Central to their argument is the role of metabolic hormones such as leptin and insulin as modulators of the developing stress system: these signals, present during sensitive developmental windows, interact with and shape the HPA axis setpoint in ways that persist into adulthood. This suggests that metabolic signals may not only shape HPA axis reactivity in acute stress situations, but that they are already associated with HPA axis regulation at early stages of its development. The authors propose that epigenetic mechanisms mediate these effects, with early-life perturbations in nutrient availability and stress hormone exposure leaving lasting marks on the expression of stress-regulatory genes [64]. This framework is directly relevant to the observations described above: if energy availability and stress regulation share common pathways from the very beginning of life, the glucose-stress relationship we describe in adulthood may itself be a downstream expression of how these systems were co-calibrated during development.

A particularly compelling, yet seemingly paradoxical, observation following severe ELA is the shift toward hypocortisolism. Severe ELA has been associated with a blunted cortisol reactivity to acute stress [61,65] and to flatter diurnal cortisol profiles [65,66]. A recent p-curve analysis supports this picture by showing that severe trauma is associated with hypocortisolism, while “milder” forms of adversity are linked to hypercortisolism [67]. Some theoretical models, such as the Adaptive Calibration Model, additionally propose a non-linear association between ELA and stress responsivity, suggesting that moderate and severe exposure may exert partially distinct physiological effects [62].

Understanding how a blunted HPA profile translates into lasting metabolic risk requires considering developmental mechanisms through which it arises. Critical windows of developmental plasticity allow the HPA axis to be programmed by the environment [68]. This is proposed to be mediated by epigenetic changes in systems sensitive to stress, altering the setpoint of the HPA axis for the remainder of the organism's life. This framework posits that metabolic disease risk emerges from the interaction of (1) a genetic predisposition, (2) epigenetic programming during sensitive developmental periods, and (3) later-life perturbations that challenge a system already set to a dysregulated baseline [69,70]. ELA may thereby contribute to a latent vulnerability that, in interaction with earlier predispositions and later environmental and behavioral factors, could increase risk for metabolic dysfunction. This complexity also explains why not every individual exposed to ELA is determined to develop metabolic dysregulations, and it further stresses the influence of moderating factors such as severity, duration, type and timing of ELA on adult pathology.

When HPA axis is impaired and fails to mount a robust, timely response to environmental challenges, the body and brain's ability to manage glucose homeostasis is compromised, since one of the key regulators of energetic flow is lacking [2]. According to the Selfish Brain Theory [40], a blunted HPA response, as observed in severe ELA, first fails to provide the necessary allocative signal (“brain-pull”) required to prioritize cerebral energy needs. One consequence of this process may be a compensatory elevation of circulating blood glucose levels, e.g., through increased energy uptake. This increases the likelihood of excess glucose accumulating in peripheral tissues. While this may fulfil the brain's needs, it comes at the long-term cost of increased energy uptake and persistent elevations in blood glucose, contributing to the development of obesity, metabolic syndrome, and ultimately type 2 diabetes [53].

**Fig. 1:**
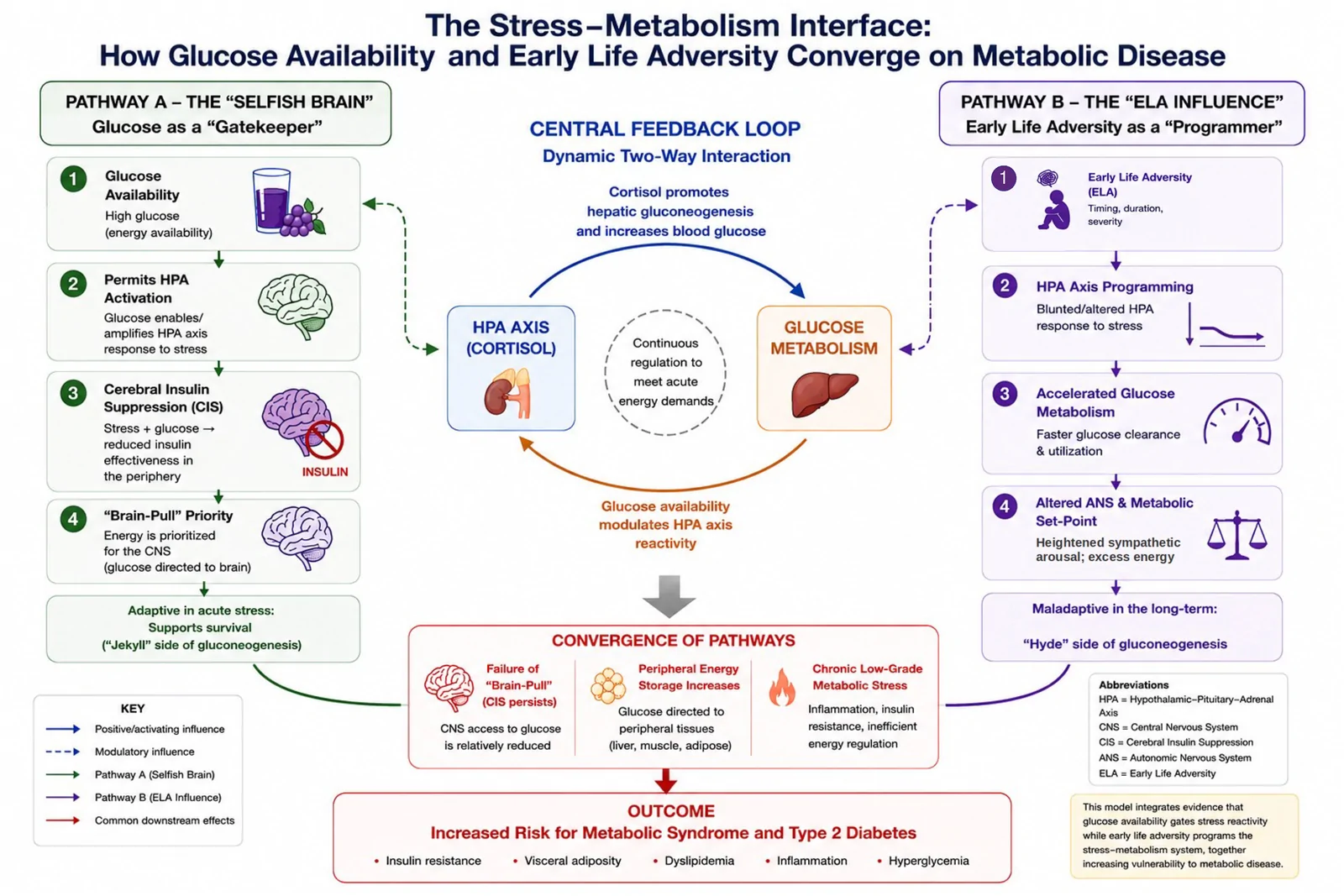
Conceptual model of glucose-dependent hypothalamic-pituitary-adrenal axis regulation, early life adversity, and long-term metabolic risk. This conceptual model illustrates the proposed interaction between glucose metabolism and stress physiology. At the center, the hypothalamic–pituitary–adrenal (HPA) axis and glucose metabolism are shown to mutually influence each other. Acute glucose availability modulates HPA axis responsivity, while cortisol and sympathetic activation influence glucose allocation and insulin regulation. Pathway A illustrates the “Selfish Brain” framework, in which glucose availability functions as a physiological gatekeeper for HPA axis activation. Under conditions of sufficient glucose availability, stress-induced activation of the HPA axis and sympathetic nervous system contributes to cerebral insulin suppression (CIS), thereby prioritizing energy allocation toward the central nervous system. Pathway B illustrates the proposed influence of early life adversity (ELA). Developmental adversity—moderated by factors such as timing, severity, chronicity, and controllability—may contribute to altered calibration of stress and metabolic systems, including blunted HPA axis responsivity and altered glucose metabolism. The convergence of these pathways (shown at the bottom) may contribute to long-term metabolic dysregulation, including obesity, metabolic syndrome, insulin resistance, and Type 2 Diabetes, by means of impaired CIS due to a lack of sufficient cortisol reactivity to life stress. The figure is conceptual and intended to summarize hypothesized associations discussed throughout the manuscript rather than depict established causal mechanisms (created with help of Claude AI).

Complementary, an impaired HPA axis and a lack of glucocorticoids fundamentally alters hepatic gluconeogenesis. This is referred to by one review as the “Dr. Jekyll and Mr. Hyde” nature of gluconeogenesis [53]: While gluconeogenesis is essential for survival during acute stress (the “Jekyll” side), chronic HPA dysregulation impairs the regulation of hepatic gluconeogenesis, which then drives the development of hyperglycemia and type 2 diabetes (the “Hyde” side). Together, these two accounts converge on the same conclusion: By impairing HPA axis regulation, ELA may contribute to long-term changes in glucose metabolism, eventually driving the system toward hyperglycemia and the development of metabolic diseases [53]. We outline this proposed mechanism in Fig. 1.

### 4.3 Glucose availability as compensatory mechanism in early life adversity?

So far, we outlined that (a) glucose administration prior to stress increases the cortisol stress response in healthy adults, and (b) ELA is associated with a blunting of the cortisol stress response, which may in turn drive maladaptive changes in glucose metabolism. Given these observations, the question arises if glucose availability could serve as a compensatory mechanism for the HPA axis dysregulation observed in individuals with a history of ELA: If the “brain-pull” is compromised by a blunted HPA axis, may elevated blood glucose represent a peripheral substitute that helps to restore cerebral energy supply in the short term, but at the long-term cost of metabolic dysregulation? We investigated this question by conducting a study testing if sugar intake could “normalize” a blunted cortisol stress response typically found in those who experienced ELA, operationalized as having perceived low maternal care in childhood [71]. We recruited 122 healthy women who were categorized into three ELA groups (very high, high, and low maternal care) based on the Parental Bonding Instrument (PBI [72]). Following an at least 8-h overnight fast, participants were randomly assigned to consume either grape juice (containing 64 g of glucose) or water approximately 30 min before being exposed to a modified group TSST [30]. We repeatedly measured salivary cortisol, salivary alpha amylase (as an ANS marker), blood glucose levels, and subjective mood ratings.

The results provided an interesting, yet unexpected picture. We initially could replicate the finding that glucose intake prior to stress increases the cortisol response in fasted individuals. Women in the juice condition showed a robust cortisol rise, while those in the water condition displayed stable or flat cortisol trajectories [71]. However, and contrary to our primary hypothesis, the extent of maternal care neither modulated the cortisol stress response, nor did it interact with glucose availability to modulate it. Two exploratory findings emerged, which are noteworthy to discuss: First, participants reporting low maternal care displayed the fastest decline in blood glucose back to baseline after sugar consumption and stress, as compared to those with high or very high maternal care. Second, a significant interaction between drink condition and maternal care was observed for salivary alpha amylase. Specifically, participants with low maternal care who consumed sugar showed higher alpha amylase levels compared to their counterparts in the water group, suggesting that glucose availability may impact autonomic activation differently depending on ELA history. Together, these results are in line with the notion that ELA is associated with alterations in glucose processing and autonomic regulation, even in a sample of otherwise healthy, young women.

The finding that glucose levels returned to baseline quicker in those with ELA may be interpreted as a faster glucose clearance, which could be a precursor to the metabolic disturbances discussed previously. If an insecure early environment signals a world of scarce or unpredictable resources, the organism may adapt by sequestering circulating glucose more rapidly into peripheral tissues [73]. Rapid glucose uptake is often driven by higher insulin levels, and research has linked ELA to increased insulin levels in psychiatric populations [71]. Over time, this chronic “pulling” of glucose into the body periphery – in light of hypocortisolism even without a channeling “brain pull”– could lead to the build-up of energy in somatic cells, contributing to obesity, metabolic syndrome and type 2 diabetes risk [41]. Finally, the study further hints at a possible mismatch between the preprogrammed physiological state and the modern environment. A system designed to efficiently store available energy for survival is ill-suited for our modern world with easy access to high-energy foods, thereby “crystallizing” the risk for metabolic syndrome if environmental circumstances shift the delicate balance of energy regulation [53]. In summary, while this study did not demonstrate the HPA axis compensation we expected, the observation of accelerated glucose metabolism in high ELA women together with the altered alpha amylase regulation provided a partial confirmation for the notion that ELA may function as a primary programmer of later-life metabolic health trajectories through stress and glucose regulation [71].

## 5 Potential future avenues

To provide a comprehensive outlook for future research, we propose three avenues that may advance the understanding of the stress-metabolism link in respect to the question of how metabolic signals shape stress regulation. First, we should expand our focus beyond the HPA axis to consistently include the ANS, which emerges in a primary and immediate regulator role for both stress regulation and energy metabolism. Much of the early work centered on cortisol and included only “accumulated” measures of ANS function, such as heart rate or salivary alpha amylase, which are influenced by both sympathetic (SNS) and parasympathetic (PNS) activity [74,75]. Yet our own work shows that adopting a multisystemic approach allows to disentangle differential influences of glucose on SNS and PNS activity in energy-dependent contexts [33,76]. In a first study [33], we investigated the effect of hyperglycemia on cardiac reactivity during slow-paced breathing (SPB), a relaxing exercise known to increase PNS activity [77]. We recruited 115 healthy adults who, after a 4-h fast, were randomly assigned to consume one of four drinks: sweet and caloric (maltodextrin and sweetener), only sweet (sweetener only), only caloric (maltodextrin only), or pure water. We measured cardiac PNS activity via the root mean square of successive differences (RMSSD) and SNS activity via the pre-ejection period (PEP) [78]. The results demonstrated a powerful and immediate effect of glucose on autonomic balance: The consumption of caloric drinks significantly altered the resting autonomic state, triggering cardiac PNS withdrawal (decreased RMSSD) and cardiac SNS activation (shortened PEP). Interestingly, despite these changes in the baseline autonomic state, the cardiac reactivity to SPB was not modulated by hyperglycemia: SPB successfully increased PNS activity and subjective relaxation across all groups, suggesting that while glucose sets the “autonomic tone” it does not necessarily hinder the system's ability to flexibly adapt to the environment. Thereby, the effects seemed to be driven by the caloric load (glucose) rather than the sensory experience of sweetness, as the non-caloric sweetener group did not show these autonomic shifts [33]. This pattern of results was replicated in a second study [76], in which 94 healthy, fasted adults were assigned to consume glucose or water prior to a standardized massage or resting intervention, both of which have previously been shown to increase PNS activity and subjective relaxation [79]. Again, glucose triggered an activation of the SNS, which did however not hinder the PNS to activate itself in response to the massage or rest intervention.

These findings provide an important empirical bridge to our previous work on ELA [71]. The fact that glucose acts as a direct stimulator of cardiac SNS activity [33,76] provides a more granular mechanistic explanation for why the SAM system may be particularly sensitive to metabolic manipulation in individuals with ELA. It suggests that the sympathetic branch may be the first to respond to metabolic changes in individuals with ELA, perhaps reflecting a programmed state of metabolic-autonomic vigilance. Thus, the current state of research highlights a critical future direction: the necessity of integrating differential markers of SNS, PNS and HPA axis activity in studies involving glucose manipulations. Because the PNS triggers insulin release and the SNS (and HPA axis) counteracts insulin to mobilize energy, evaluating only one system provides an incomplete and potentially misleading picture of biological system integrity.

Second, and along the line of advocating the adoption of a multisystemic perspective, future studies should investigate whether other metabolic hormones, such as insulin, ghrelin, leptin or neuropeptide Y, modulate the stress response. The effect of other metabolic signals beyond glucose on stress regulation are widely understudied in healthy populations to date [35,80]. Expanding this literature through pharmacological approaches and careful consideration of potential differential peripheral and central actions of metabolic hormones, as documented for instance for insulin [81], will be important to disentangle the causal contributions of these highly correlated effectors.

Third, moving beyond controlled laboratory settings to investigate how energy metabolism and food intake shape stress regulation in daily life. One the one hand, this could mean moving beyond standardized stress scenarios that target the individual to more natural, socially driven scenarios in group settings that target questions of how negative affect triggered by stress is coped with in “real-life scenarios” that allow for compensation by both social as well as “food-related” means. Since emotion regulation and social functioning is deeply tied to cardiac PNS activation [82] which is in turn affected by, and a trigger for metabolic signals, exploring how hyperglycemia impacts the cardiac response to social stimuli may be an important and promising frontier for understanding stress-related and metabolic disorders. On the other hand, this could mean moving out of the laboratory and into the field to determine whether the effects observed in controlled settings actually translate to stress regulation in daily life. Findings linking the intake of comfort food to dampened stress responses already questions whether glucose intake may be associated with increased cortisol reactivity to stress ubiquitously [83]. In line with that, a recent ambulatory study found that higher circulating glucose levels buffered stress in daily life by showing that higher glucose was associated with a relative decrease in subjective stress ratings [35]. As such, the glucose-stress link may take different shapes and forms, dependent on the context, the determinants of which should systematically be tested in the future. Importantly, across all three steps, it will be important to consider not only studying how metabolic factors shape stress regulation in the healthy individuals, but also to include populations with varying degrees of stress-related or metabolic vulnerabilities and observe the co-regulation of the stress and the metabolic system not only cross-sectionally, but in a longitudinal manner. Together, we deem that these contributions would be promising avenues with high prospects of bettering our understanding of the stress-metabolism interface.

## 6 Conclusion

Research over the past decades increasingly suggested that glucose availability is not merely an outcome of the acute stress response but also a regulator of stress responsivity itself. Building on the original work by Clemens Kirschbaum, a line of research emerged that consistently showed that glucose administration can enhance cortisol responses to acute stressors, whereas fasting may attenuate it. The Selfish Brain Theory [40] offers a possible framework for interpreting these findings by conceptualizing stress systems as mechanisms involved in cerebral energy allocation. Multiple pathways may contribute to the often-observed increased risk for metabolic disease following stress in sensitive developmental period. Here, we particularly discussed the “Jekyll and Hide” sides of gluconeogenesis [53]. Overall, the “next frontier” for metabolism-stress research could lie in acknowledging more prominently that glucose administration does not simply boost the HPA axis; it actively reconfigures all of the autonomic and endocrine systems depending on the acute energy needs. Moving beyond cross-sectional laboratory studies of isolated individuals toward multisystemic, naturalistic approaches will be essential to capture how genetic predispositions, epigenetic programming, and later-life experiences jointly determine the vulnerability to the metabolic disturbances that so frequently follow ELA.

## Declaration of generative AI and AI-assisted technologies in the manuscript preparation process

AI-assisted technology (Google Scholar, notebook.lm, Gemini, Claude) was used for literature search and summaries as well as for structuring ideas. However, all literature selection, interpretation of findings, scientific synthesis, critical evaluation, and manuscript writing were independently performed and verified by the authors, who take full responsibility for the final content of the published article.

## CRediT authorship contribution statement

**Jens C. Pruessner:** Conceptualization, Formal analysis, Project administration, Supervision, Writing – original draft, Writing – review & editing. **Maria Meier:** Conceptualization, Methodology, Project administration, Supervision, Writing – review & editing.

## Declaration of competing interest

The authors have nothing to disclose.

## Footnotes

1 prominently accelerated by the introduction of psychosocial stress paradigms like the Trier Social Stress Test, a major contribution of Clemens Kirschbaum to our field [9].
